# Comparison of Capsule Endoscopy Findings to Subsequent Double Balloon Enteroscopy: A Dual Center Experience

**DOI:** 10.1155/2015/438757

**Published:** 2015-09-01

**Authors:** Amandeep S. Kalra, Andrew J. Walker, Mark E. Benson, Anurag Soni, Nalini M. Guda, Mehak Misha, Deepak V. Gopal

**Affiliations:** ^1^Division of Hospital Medicine, Department of Medicine, University of Wisconsin School of Medicine and Public Health, 1685 Highland Avenue, MFCB 3116, Madison, WI 53705, USA; ^2^Division of Gastroenterology and Hepatology, Department of Medicine, University of Wisconsin School of Medicine and Public Health, 1685 Highland Avenue, MFCB 4223, Madison, WI 53705, USA; ^3^Division of Gastroenterology & Hepatology, University of Wisconsin School of Medicine & Public Health, GI Associates-Aurora St. Luke's Medical Center, 2801 W Kinnickinnic River Pkwy, Suite 1030, Milwaukee, WI 53215, USA; ^4^Department of Gynecology, Manipal College of Medical Sciences, Pokhara 33700, Nepal

## Abstract

*Background*. There has been a growing use of both capsule endoscopy (CE) and double balloon enteroscopy (DBE) to diagnose and treat patients with obscure gastrointestinal blood loss and suspected small bowel pathology. *Aim*. To compare and correlate sequential CE and DBE findings in a large series of patients at two tertiary level hospitals in Wisconsin. *Methods*. An IRB approved retrospective study of patients who underwent sequential CE and DBE, at two separate tertiary care academic centers from May 2007 to December 2011, was performed. *Results*. 116 patients were included in the study. The mean age ± SD was 66.6 ± 13.2 years. There were 56% males and 43.9% females. Measure of agreement between prior capsule and DBE findings was performed using kappa statistics, which gave kappa value of 0.396 with *P* < 0.001. Also contingency coefficient was calculated and was found to be 0.732 (*P* < 0.001). *Conclusions*. Our study showed good overall agreement between DBE and CE. Findings of angioectasia had maximum agreement of 69%.

## 1. Introduction

After the introduction of double balloon enteroscopy (DBE) and capsule endoscopy (CE) in years 2000 and 2001, respectively, there has been significant improvement in the management of obscure gastrointestinal bleeding (OGIB) [1, 2]. OGIB accounts for 5 percent of total GI bleeding, but it results in multiple transfusions and repeated hospitalization [2, 3]. Until the advent of wireless capsule endoscopy (CE) and balloon-assisted deep bowel enteroscopy in the last fifteen years, this had been a difficult area to evaluate and intervene upon, and in many cases the etiology of small bowel bleeding remained undiagnosed using standard endoscopic and imaging modalities. Wireless CE is generally the test of choice for patients with OGIB who have undergone preceding upper endoscopy and colonoscopy because of its capability to examine the entire small bowel with reasonable accuracy and it is relatively noninvasive and well tolerated. Additionally, most capsule endoscopy studies (70–80%) are performed for obscure small bowel bleeding [4]. Capsule endoscopy is limited by its inability to biopsy the lesion or to do any therapeutic interventions [2]. For this reason, balloon-assisted deep enteroscopy is primarily performed in conjunction with CE. Despite being more invasive than CE, one method of balloon-assisted deep enteroscopy, DBE, has been shown to be clinically impactful. For instance, one study demonstrated that DBE could detect a source of GI bleeding in nearly 80% of cases and clinically control bleeding in 65% of cases [5].

Prior investigations comparing the diagnostic yield between CE and DBE have shown comparable diagnostic yield but have been limited by small sample size. The purpose of this study is to compare and correlate sequential CE and DBE findings of the small bowel in a large series of patients at two tertiary level hospitals in Wisconsin.

## 2. Methods

### 2.1. Patients

A retrospective review at two tertiary medical centers was performed for all patients with OGIB who underwent CE and sequential DBE from May 2007 to December 2011. Institutional Review Boards at both University of Wisconsin Hospital and Clinics and Aurora St. Lukes Medical Center approved the study in which 116 patients were identified. All patients suffered from OGIB and had undergone upper gastrointestinal endoscopy and colonoscopy without the discovery of any identifiable lesion. OGIB was defined as overt bleeding or recurrent fecal occult bleeding with anemia of unknown origin. DBE was preceded by CE within 1 year in all cases. The total number of patients in this study was 116, out of which 65 (56%) were males and 51 (43.9%) females with a mean age of 66.6 ± 13.2 years.

### 2.2. CE

In CE the video capsules (Medtronic, Duluth, GA, USA) were propelled through the gastrointestinal tract by peristalsis. Prokinetics were not employed. Sensors were activated on patient's abdomen and connected to mobile recording system. Later during the same day the equipment was removed and data were uploaded onto a computer system. The technical procedures and evaluation of capsule images have been previously described [6]. This was interpreted by an experienced gastroenterology staff at University of Wisconsin Hospital and Aurora St. Lukes Medical Center. All reviewers had reviewed >10 capsule examinations prior to initiation of the study. The locations of the lesions in the small bowel were determined by means of transit times.

### 2.3. DBE

The DBE system (Fujifilm Medical System, Stanford, CT, USA) is a video endoscope that consists of a flexible overtube and a balloon controller. Double balloon endoscope has a 200 cm working length and 145 cm overtube, with a diameter, which varies from 8.5 mm within the working length of the tube to 12.5 mm at the overtube. DBE was done either anterogradely (orally) or retrogradely (per rectum) or both. Patients were instructed not to eat anything after the midnight before the procedure. For retrograde DBE patients underwent bowel preparation the night before the procedure. Diagnostic biopsies or therapeutic procedures were performed via a 2.2 mm diameter-working channel and the site of most distal advancement was tattooed. DBE was performed under general anesthesia. In anterograde and retrograde DBE scope is inserted orally and rectally, respectively, and advanced in repetitive cycles of inflation and deflation. DBE followed CE in all cases. The length of small bowel investigated was estimated endoscopically during introduction and withdrawal of the scope. DBE was performed by 4 different endoscopists (DVG, MB, AS, and NG), each had performed >10 prior exams. The tube is later withdrawn with inflation and deflation techniques.

### 2.4. Interpretation of Findings/Data Analysis

All cases were examined for findings that could explain blood loss. These included presence of active bleeding source or culprit lesion such as ulceration (NSAID, Crohn's disease, and ischemic ulceration), arteriovenous malformations (AVMs), polyp, erosion, diverticulum, tumor, or Dieulafoy lesion. A positive study was defined as the presence of one of the above features. The CE findings were directly compared with the DBE findings for agreement with the DBE findings acting as the gold standard. All DBE cases were also reviewed for therapeutic intervention.

## 3. Results and Discussion

### 3.1. Results

**Figure 1:**
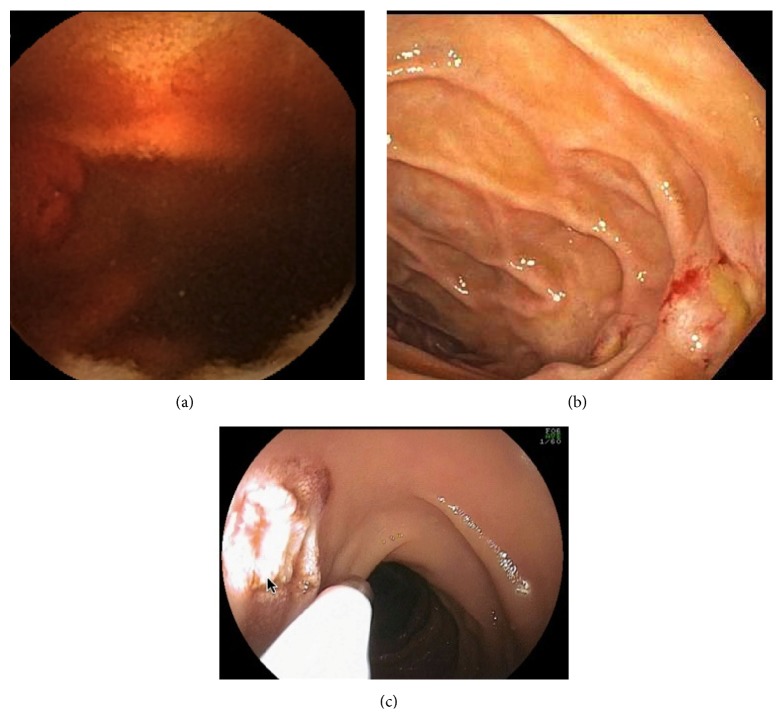
(a) 63-year-old male with bleeding jejunal AVM on CE. (b) 63-year-old male with corresponding AVM on DBE. (c) 63-year-old male with corresponding AVM treated with TouchSoft coagulation (Genii Inc., St. Paul, MN, USA) therapy with DBE.

**Figure 2:**
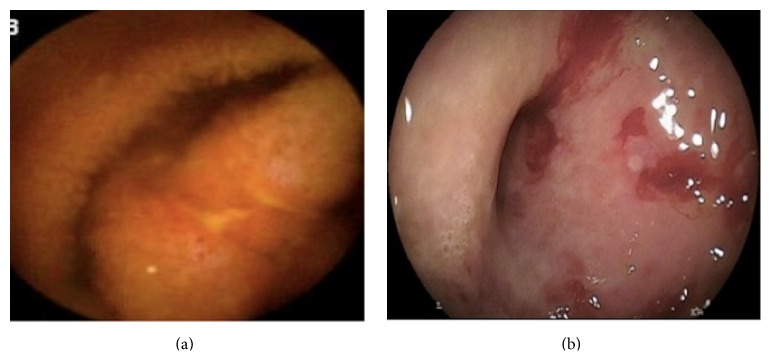
(a) CE of small bowel uclerations in distal jejunum/proximal ileum in 43-year-old female patient with severe iron deficiency anemia and abdominal pain. (b) Corresponding DBE in above patient ulcerated stricture consistent with a small bowel Crohn's disease involving distal jejunum/proximal ileum.

116 patients were included in the study. The mean age ± SD was 66.6 ± 13.2 years. There were 56% males and 43.9% females, respectively.  Table 1 shows the cross tabulation of CE and DBE with various findings. Measure of agreement between prior capsule and DBE findings was performed using kappa statistics, which gave kappa value of 0.396 (*P* < 0.001). Also contingency coefficient was calculated and was found to be 0.732 (*P* < 0.001), indicating good overall agreement. AVMs had best agreement (69%; Figures 1(a), 1(b), and 1(c)), followed by ulcer disease (60%; Figures 2(a) and 2(b)).

Interestingly, in our study, 42% of normal findings were found to have AVMs on DBE. The two main indications of performance in both studies were anemia and bleeding (Table 2).  Table 1 shows cross tabulation for individual indication of anemia and bleeding between DBE and CE. Contingency coefficient was calculated between CE and DBE, after splitting the number of cases with anemia and bleeding, which was found to be 0.736 and 0.772 indicating good agreement with either indication.

### 3.2. Discussion

In our study we found that there is a high correlation between CE and DBE for small bowel angiodysplasias/AVMs at 69% followed by normal findings. These findings were similar to a retrospective study done by Marmo et al., which found a good agreement for vascular or inflammatory lesions but not for polyps or neoplasia [7]. In a prospective study done by Matsumoto et al. in which DBE was done prior to CE and CE observer was blinded to the results, authors found good concordance in diagnosing OGIB between the two modalities, but DBE was superior to CE in diagnosing small intestine polyps [8]. A meta-analysis from 2013 that looked in 12 studies, with 712 patients, who have undergone CE or DBE for OGIB found that CE and DBE had similar diagnostic capabilities for vascular, ulcerative, neoplastic, and inflammatory lesions [9]. In the same meta-analysis, it was found that CE had significantly better diagnostic yield for fresh blood or clot whereas DBE has better diagnostic yield for small bowel diverticular bleed.

Kameda et al. demonstrated a rate of agreement between CE and DBE that was 50% of all cases [10], whereas we showed that the total rate of agreement was 64.6%. In a previous study it was found by authors that CE has excellent negative predictive value for lesions in small intestine, except for polyps in which DBE has better negative predictive value than CE [11]. In our study the overall correlation for small bowel lesions other than angiodysplasias/AVMs, ulcer disease, or normal findings was suboptimal. One of the challenges with CE is the indefinite and nondiagnostic findings. In our study, CE showed indefinite abnormal results in 2.5% of patients. In our study no AVMs were detected in 14 cases of CE, but AVMs were found on subsequent DBE, out of which 12 cases had normal findings on previous CE. In our study 42% of all the normal CE studies had findings on subsequent DBE potentially accounting for the patient symptoms.

In a previous single blind prospective study, 32 patients with OGIB underwent CE followed by DBE determined that CE detected abnormal findings in 90.6% patients as compared to DBE which detected abnormal findings in 65.6% [10]. We similarly showed that more abnormal findings were seen on CE than DBE. In a meta-analysis done from 2007, which included 8 studies with 277 patients, the authors noted no significant difference in diagnostic yield of CE and complete DBE (anterograde and retrograde DBE), but diagnostic yield of CE was found to be higher than either anterograde or retrograde approach alone [12]. In another meta-analysis done in 2008, which looked in 11 studies, comparing DBE and CE concluded that yield of CE and DBE in finding pathology of small bowel was similar [13].

## 4. Conclusions

Our study confirms that CE and DBE share a complementary role in diagnosing OGIB. CE that is performed first is useful to direct the route of balloon-assisted enteroscopy when the indication is OGIB or anemia particularly when the findings are AVMs, giving good agreement seen between the two procedures in our study. As discussed above, AVMs demonstrate best agreement, which can be explained from the fact that most common indications for both studies are anemia or OGIB with AVM being major cause of either indication. Previous studies have shown that when CE and DBE are done together this provides a better diagnostic yield and better directed therapy [14]. Interestingly, in our study, 42% of normal findings were found to have angiodysplasias/AVMs on DBE, which would argue towards proceeding with DBE in patients with normal findings on CE or even proceeding with DBE without conducting CE if the indication is anemia or bleeding with high suspicion of AVM. The merits of our study over other previous studies are that it is a large dual center study with a large number of patients' population with two only indications for CE and DBE being anemia or OGIB. The main limitation is the retrospective nature of our study and the discrepancy between AVMs and any other findings.

## Figures and Tables

**Table 1:** Correlation between CE and DBE findings.

*N* = number of corresponding findings		DBE findings							Total
		AVM	Erosions	Normal	Polyp	Stricture	Ulcer	Others	
Capsule	AVM	55	0	11	2	0	0	1	69
	Erosions	1	1	1	0	0	0	0	3
	Normal	12	0	13	0	1	2	0	28
	Polyp	0	0	2	0	0	0	0	2
	Stricture	0	0	1	0	0	0	0	1
	Ulcer	0	0	4	0	0	6	0	10
	Others	1	0	1	0	0	0	1	3
	Total	69	1	33	2	1	8	2	116

**Table 2:** CE and DBE findings correlated based on findings (anemia versus OGIB).

Indication	DBE findings							Total
	AVM	Erosion	Normal	Polyp	Stricture	Ulcer	Others	
Anemia								
Prior CE								
AVM	29		3		0	0	0	32
Erosion	1		1		0	0	0	2
Normal	7		9		1	1	0	18
Polyp	0		1		0	0	0	1
Stricture	0		1		0	0	0	1
Ulcer	0		2		0	4	0	6
Others	1		1		0	0	1	3
Total	38		18		1	5	1	63

Bleeding								
Prior CE								
AVM	26	0	8	2		0	1	37
Erosion	0	1	0	0		0	0	1
Normal	5	0	4	0		1	0	10
Polyp	0	0	1	0		0	0	1
Others	0	0	2	0		2	0	4
Total	31	1	15	2		3	1	53
